# Evidence of a Causal Relationship Between Vitamin D Status and Risk of Psoriasis From the UK Biobank Study

**DOI:** 10.3389/fnut.2022.807344

**Published:** 2022-07-25

**Authors:** Yan Zhang, Danrong Jing, Guowei Zhou, Yi Xiao, Minxue Shen, Xiang Chen, Hong Liu

**Affiliations:** ^1^Department of Dermatology, Xiangya Hospital, Central South University, Changsha, China; ^2^Department of Respiratory Medicine, Xiangya Hospital, Central South University, Changsha, China; ^3^Hunan Key Laboratory of Skin Cancer and Psoriasis, Xiangya Hospital, Changsha, China; ^4^Hunan Engineering Research Center of Skin Health and Disease, Xiangya Hospital, Changsha, China; ^5^Department of Social Medicine and Health Management, Xiangya School of Public Health, Central South University, Changsha, China; ^6^Xiangya Clinical Research Center for Cancer Immunotherapy, Central South University, Changsha, China; ^7^National Clinical Research Center for Geriatric Disorders, Xiangya Hospital, Central South University, Changsha, China

**Keywords:** psoriasis, vitamin D, causality, MR, BMI

## Abstract

**Background:**

Plenty of observational studies suggested that vitamin D concentrations were associated with psoriasis, but the causality of this relationship was elusive.

**Objective:**

To investigate the causal relationship between vitamin D and psoriasis.

**Methods:**

Cox proportional hazard model was used to investigate the association between vitamin D status and psoriasis in a prospective cohort study from UK Biobank. Single nucleotide polymorphisms (SNPs) that are strongly associated with circulating 25OHD were constructed as instrumental variables in Mendelian randomization (MR) to determine the causality between vitamin D and psoriasis.

**Results:**

During a median follow-up of 10.99 years, we identified 2,856 participants with incident psoriasis. The prospective cohort study demonstrated individuals with 25OHD deficiency (< 25 nmol/L) at baseline were associated with approximately 20% increased risk of incident psoriasis in different categories of sex, age, and body mass index (BMI) after adjusting for covariates. The largest effect size was observed in the obese group (BMI > 30 kg/m^2^), as 25OHD deficiency presented with 30% additional risk of incident psoriasis compared to those with 25OHD > 50 nmol/L (HR = 0.701; 95% CI: 0.583–0.843; *p* < 0.001). Additionally, 69 independent SNPs associated with circulating 25OHD level were selected for the MR analysis, and the result suggested that genetically predicted one standard deviation (SD) increment in log-transformed 25OHD was associated with 24% decreased risk of psoriasis (OR = 0.76; 95% CI: 0.60–0.98, *p* = 0.020).

**Limitations:**

The association of 25OHD and severity of psoriasis could not be estimated in the current study.

**Conclusion:**

The combined prospective and MR analysis additionally provided evidence that the epidemiologically and genetically determined level of 25OHD conferred an increased risk of psoriasis.

## Capsule Summary

The prospective cohort study indicated that circulating 25OHD was inversely associated with the risk of incident psoriasis, particularly in subgroups of obesity. Mendelian randomization analysis further confirmed the causal relationship between 25OHD and psoriasis genetically. Randomized controlled trials and real-world evidence are warranted to determine the efficacy of systemic management of 25OHD in the prevention of psoriasis, especially for obese individuals with 25OHD deficiency.

## Introduction

Psoriasis is a common chronic, immune-mediated inflammatory skin disorder that affects approximately 2% of the world population, and markedly impaired quality of life (1, 2). It is characterized by keratinocyte hyperproliferation and dysregulated T-helper 17 immune response with markedly altered inflammatory cytokine profiles (3). Although its etiology has not yet been fully elucidated, genetic and environmental factors are thought to play a critical role in the pathogenesis of psoriasis.

To date, a large number of treatments have been used for psoriasis, including phototherapy, topical, and oral medications, conventional systemic drugs, and small molecules, and also biologics (4). The topical vitamin D analogs, either alone, or being adjunctive treatment of corticosteroids, is one of the most well-known options for the management of psoriasis (5). The compound 25-hydroxy vitamin D (25OHD) exerts immunomodulatory and anti-inflammatory effect by modulating the innate and adaptive immune system, blocking proliferation, and boosting differentiation and maturation of keratinocyte (6). It can ameliorate T-cell proliferation and promote the differentiation of regulatory T cells (Tregs), and it also can regulate macrophage response and prevent proinflammatory cytokines release from macrophages (7). And vitamin D treated *in vitro* differentiated DCs express decreased levels of costimulatory molecules and increased levels of inhibitory receptors. Moreover, they secrete lower amounts of proinflammatory and higher amounts of anti-inflammatory cytokines (8). Therefore, the deficiency of 25OHD may contribute to the development of psoriasis *via* the reduction in immunomodulatory, anti-inflammatory, and antiproliferative activities. However, the causal relationship between 25OHD and risk of psoriasis and the disease severity of psoriasis has not been truly and consistently determined from previously retrospective and cross-sectional studies (9–11).

To overcome the limitations of cross-sectional studies and resolve inconsistencies, we conducted a prospective cohort study in a large European adult population from the UK Biobank to estimate the association of serum 25OHD levels with the risk of psoriasis. We then performed a Mendelian randomization (MR) analysis to genetically uncover the causal relationship between 25OHD and psoriasis.

## Materials and Methods

### Study Design and Participant

The UK Biobank is a large-scale prospective cohort study with more than 500,000 participants aged 40–69 from 22 medical centers throughout the United Kingdom, recruited between 2006 and 2010 (12). The data used in the current study was from the UK Biobank that had received an approval from the North West Multi-Center Research Ethics Committee and informed consents from all individuals prior to participation. Medical history was provided for each participant at baseline assessment through interviews, touchscreen questionnaires, and physical measurements. The participants also donated blood and urine samples for future analysis and agreed to be followed up through linkage to their health records. Analyses in this article were restricted to individuals with complete information on both 25OHD and genetic polymorphisms.

### Ascertainment of Outcome

Based on the clarifications from the UK Biobank, diagnosis of psoriasis was mainly confirmed using hospital inpatient records obtained from the Hospital Episode Statistics for England, Scottish Morbidity Record data for Scotland, and the Patient Episode Database for Wales. Additional cases were complemented through linkage to self-report, primary care, and death register data. The international classification of diseases (ICD) coding system was used to record the diagnosis of psoriasis as well.

### Assessment of Exposure

The biological sample was obtained from each participant at the initial assessment center visit. Serum 25OHD level was determined by chemiluminescence immunoassay with high sensitivity and precision (13).

Genetic instruments of independent common single nucleotide polymorphisms (SNPs) that being closely associated with circulating 25OHD levels were extracted from a meta-analysis of genome-wide association studies (GWAS), including 401,460 white British participants from the UK Biobank and another dataset of 42,274 Europeans (14). Finally, 69 SNPs with significant genome-wide 25OHD level were selected as genetic instruments (*p* < 6.6 × 10^–9^). Only common SNPs among the conditionally independent SNPs were included for the MR analysis to ensure that our instruments were truly in linkage equilibrium, since the *r*^2^ as a metric of linkage disequilibrium (LD) is less accurate for rare variants. It was estimated that these SNPs collectively explained about 3.1% of variance of circulating 25OHD levels, and the variance explained for a given SNP was calculated using the formula: variance explained = 2β 2 *f* (1–*f*), where β and *f* denote the effect of the SNP on 25OHD level and the MAF, respectively. GWAS summary data of psoriasis from MRC-IEU, OpenGWAS data^1^ were used for MR analysis (15), after excluding instrumental variables with significant genome-wide significance (*p* < 5 × 10^–8^) and minor allele frequency (MAF) < 5% to prevent the effects of rare variants and horizontal pleiotropy. Finally, we calculated the F statistics of all instrumental variables included in MR analyses to confirm that the F statistics of all SNPs included in the study were more than 10 (16).

### Mendelian Randomization Analysis

To investigate whether there is a causal relationship between circulating 25OHD level and risk of psoriasis, a main analysis was conducted to estimate the effect of one increment in SD of natural log-transformed 25OHD on psoriasis using the inverse-variance weighted (IVW) method as previously described (17). The IVW method weighed the effect of each instrumental variable on psoriasis susceptibility by its effect on 25OHD using the Wald ratio method, and then combined these individual MR estimates into a random effect inverse-variance meta-analysis (18).

In addition, three additional methods were applied to control for pleiotropy (weighted median, MR-Egger, and mode-based estimate) and to compare the respective MR estimates. The MR-Egger method can evaluate the potential pleiotropy in MR analysis, and offset its effect by intercepts (19). The weighted median method uses weighted median of ratios of all instrumental variables, and can tolerate the weight of invalid SNPs up to 50% (20). Mode-based estimate is a method to obtain causal effect estimate from multiple genetic instruments, which can allow potential pleiotropy in majority of instrumental variables (21). In order to reduce the effect of outliers, robust regression with penalized parameter was used in the MR Egger and IVW methods.

Sensitivity analyses were conducted by excluding SNP variants associated with potential confounders. The PhenoScanner database was queried for each 25OHD-related instrumental SNP to identify genetic variants (associating variants) related to GWAS traits that are potential confounders or could introduce horizontal pleiotropy in the exposure–outcome association.

### Statistical Analysis

We assessed the association of baseline circulating 25OHD level with the risk of incident psoriasis using Cox proportional hazard models, and estimated the hazard ratios (HRs) and 95% confidence intervals (CIs) after adjusting for covariates. The basically adjusted model included age, sex, and body mass index (BMI); and the fully adjusted model additionally included income, education, smoking status, and 25OHD supplements.

The dose–response relationship between 25OHD and incident psoriasis was plotted using cubic splines. We then standardized the serum 25OHD levels to a normal distribution and estimated the HR corresponding one SD increment. We also divided the 25OHD levels into quartiles (25th, 50th, and 75th) as well as clinical categories (deficient: < 25 nmol/L, insufficient: 25∼50 nmol/L, optimal: > 50 nmol/L). Subsequently, stratified analyses were conducted based on sex, age group, and BMI.

The cohort data was analyzed by R Version 3.6.3. The GWAS summary data was extracted by R package “TwoSampleMR” and MR, and sensitivity analyses were performed using R package “Mendelian Randomization.” A *p*-value less than 0.05 was considered of statistical significance (two sided). We also applied the global test, outlier test, and distortion test using R package “MR-PRESSO.”

## Results

### Selection and Baseline Characteristics of Participants

In the current study, 429,681 participants were included and eligible for the analysis after excluding 9,953 participants with prevalent psoriasis, 8,320 with no genetic data, and 54,551 with missing data on serum 25OHD information (Supplementary Figure 1). We identified 2,856 participants with incident psoriasis during a median follow-up period of 10.99 years. Baseline characteristics including age, sex, household income, background of education, smoking status, BMI, serum 25OHD concentration, and use of vitamin D supplements are presented in Table 1.

**TABLE 1:** Characteristics of participants from the UK Biobank.

Covariate	Total (*N* = 429,681)	Psoriasis (*N* = 2,856)	No Psoriasis (*N* = 426,825)	*p* ^b^
Age at baseline (years), mean (*SD*)	56.50 (8.12)	57.17 (7.93)	56.49 (8.12)	<0.001
Age category (years), *N* (%)				<0.001
<50	101,816 (23.7)	569 (19.9)	101,247 (23.7)	
50–59	142,255 (33.1)	969 (33.9)	141,286 (33.1)	
> 60	185,610 (43.2)	1,318 (6.1)	184,292 (43.2)	
Sex, *N* (%)				0.137
Female	230,896 (53.7)	1,495 (52.3)	229,401 (53.7)	
Male	198,785 (46.3)	1,361 (47.7)	197,424 (46.3)	
Average total household income before tax (€), *N* (%)				<0.001
Less than 18,000	82,199 (19.1)	689 (24.1)	81,510 (19.1)	
18,000–30,999	93,161 (21.7)	630 (22.1)	92,531 (21.7)	
31,000–51,999	96,226 (22.4)	588 (20.6)	95,638 (22.4)	
52,000–100,000	75,872 (17.7)	433 (15.2)	75,439 (17.7)	
Greater than 100,000	20,262 (4.7)	95 (3.3)	20,167 (4.7)	
Unknown	61,961 (14.4)	421 (14.7)	61,540 (14.4)	
Education, *N* (%)				<0.001
College or university degree	139,999 (32.6)	824 (28.9)	139,175 (32.6)	
Professional qualifications	50,538 (11.8)	359 (12.6)	50,179 (11.8)	
A Levels/AS levels or equivalent	47,823 (11.1)	265 (9.3)	47,558 (11.2)	
O Levels/GCSEs or equivalent	113,917 (26.5)	748 (26.2)	113,169 (26.5)	
None of the above	76,980 (17.9)	657 (23.0)	76,323 (17.9)	
Smoking status, *N* (%)				<0.001
Never	235,288 (54.8)	1,253 (43.9)	234,035 (54.9)	
Previous	148,358 (34.6)	1,114 (39.0)	147,244 (34.5)	
Current	43,925 (10.2)	473 (16.6)	43,452 (10.2)	
Unknown	1,683 (0.4)	13 (0.5)	1,670 (0.4)	
BMI (kg/m^2^), mean (SD)	27.40 (4.78)	28.37 (5.24)	27.39 (4.77)	<0.001
BMI category, *N* (%)				<0.001
Normal (<25)	142,528 (33.3)	744 (26.1)	141,784 (33.3)	
Overweight (25∼30)	181,802 (42.5)	1,234 (43.3)	180,568 (42.5)	
Obesity (> 30)	103,731 (24.2)	869 (30.5)	102,862 (24.2)	
Vitamin D concentrations (nmol/L), Mean (*SD*)	48.26 (19.69)	46.68 (20.04)	48.27 (19.69)	<0.001
Vitamin D concentrations (nmol/L)^a^, *N* (%)				<0.001
12.7–32.6	106,657 (24.8)	812 (28.4)	105,845 (24.8)	
32.6–46.8	107,675 (25.1)	703 (24.6)	106,972 (25.1)	
46.8–62.0	107,454 (25.0)	697 (24.4)	106,757 (25.0)	
62.0–104.0	107,895 (25.1)	644 (22.5)	107,251 (25.1)	
Vitamin D category (nmol/L), *N* (%)				<0.001
Deficient (<25)	54,306 (12.7)	460 (16.1)	54,306 (12.7)	
Insufficient (25∼50)	183,631 (42.7)	1,210 (42.4)	182,421 (42.7)	
Optimal (> 50)	191,284 (44.6)	1,168 (41.5)	190,098 (44.6)	
Vitamin D supplements, *N* (%)				0.197
No	421,883 (98.2)	2,795 (97.9)	419,088 (98.2)	
Yes	7,798 (1.8)	61 (2.1)	7,737 (1.8)	

*^a^The data were divided by quartiles.*

*^b^Continuous data was presented as mean ± standard deviation, and between-group difference was tested using analysis of variance. Categorical data were presented as number (%), and the between-group difference was tested using chi-squared test. p < 0.05 was considered statistically significant for all tests.*

### Association of Observed Circulating 25OHD Levels and Incident Psoriasis

The association between observed circulating 25OHD level and risk of incident psoriasis was estimated in Cox models by including covariates (Table 2). One SD increment in 25OHD level was associated with 7.0% decreased risk of incident psoriasis (HR = 0.930; 95% CI: 0.896–0.965; *p* < 0.001) in basically adjusted model which included age, sex, and BMI. Similar effect (HR = 0.938; 95% CI: 0.904–0.974; *p* = 0.001) was observed in the fully adjusted model which additionally included household income, education, smoking status, and 25OHD supplements.

**TABLE 2:** Association of vitamin D concentrations with incident psoriasis.

			Model 1^a^		Model 2^b^	
Vitamin D concentration	N	Person-years	HR (95% CI)	*p*	HR (95% CI)	*p*
Per SD in concentration	2,856	4,674,462	0.930 (0.896–0.965)	<0.001	0.938 (0.904–0.974)	0.001
**Quartiles**						
12.7–32.6	812	1,160,748	Ref		Ref	
32.6–46.8	703	1,171,944	0.865 (0.781–0.957)	0.005	0.882 (0.797–0.977)	0.016
46.8–62.0	697	1,168,501	0.874 (0.789–0.968)	0.010	0.896 (0.809–0.993)	0.037
62.0–104.0	644	1,173,269	0.827 (0.744–0.919)	<0.001	0.841 (0.757–0.935)	0.001
			*p*_trend_ = 0.002		P_trend_ = 0.010	
**Category**						
Deficient (<25)	460	596,195	Ref		Ref	
Insufficient (25∼50)	1,210	1,998,285	0.791 (0.710–0.882)	<0.001	0.814 (0.731–0.907)	<0.001
Optimal (> 50)	1,186	2,079,982	0.770 (0.690–0.860)	<0.001	0.795 (0.712–0.887)	<0.001
			*p*_trend_ < 0.001		*p*_trend_ < 0.001	

*^a^Model 1 was adjusted for age, sex, and BMI.*

*^b^Model 2 was additionally adjusted for income, education, smoking status, and vitamin D supplements.*

Then, the participants were divided into four categories by quartiles of serum 25OHD concentrations and three categories by clinical cut-offs. The risk of incident psoriasis decreased with increasing 25OHD concentration (*p*_trend_ = 0.002), and individuals in the highest quartile of 25OHD concentration had 17.3% decreased risk of incident psoriasis (HR = 0.827; 95% CI: 0.744–0.919; *p* = 0.002) compared to those in the lowest quartile, after adjustment for age, sex, and BMI. Similar effect was also observed in participants with higher 25OHD levels after additional adjustment for income, education, smoking status, and 25OHD supplements (*p*_trend_ = 0.010). Moreover, compared to 25OHD deficiency, the risk of psoriasis was decreased by 18.6% (HR = 0.814; 95% CI: 0.731–0.907; *p* < 0.001) and 20.5% (HR = 0.795; 95% CI: 0.712–0.887; *p* < 0.001) in participants with insufficient and sufficient 25OHD levels, respectively, in the fully adjusted model.

### Subgroup Analysis for the Association of Observed 25OHD Level With Psoriasis

Stratified analysis based on sex (Supplementary Table 1), age group (Supplementary Table 2), and BMI (Table 3) categories were estimated by HR in fully adjusted model to determine the relationship between serum 25OHD concentration and risk of incident psoriasis. Overall, per one SD decrease of 25OHD level was associated with increased risk of psoriasis. The prospective cohort study demonstrated individuals with 25OHD deficiency (<25 nmol/L) at baseline were associated with approximately 20% increased risk of incident psoriasis when compared to individuals with optimal 25OHD (>50 nmol/L) in different categories of sex, age, and BMI after adjusting for covariates. The largest effect size was observed in the obese group (BMI > 30 kg/m^2^), as 25OHD deficiency presented with 30% additional risk of incident psoriasis compared to those with 25OHD > 50 nmol/L (HR = 0.701; 95% CI: 0.583–0.843; *p* < 0.001, Table 3). However, the interaction effect between BMI and 25OHD was calculated, and no interaction effects were observed between BMI and 25OHD (Supplementary Table 3). Effect size observed in male and female groups were similar. And in the age stratified analysis, the effect seems more obvious in the population less than 50 years old.

**TABLE 3:** Association of vitamin D concentrations with incident psoriasis in different BMI categories.

Vitamin D concentration, nmol/L	Normal (BMI < 25)				Overweight (25 < BMI < 30)				Obesity (BMI > 30)			
	*N*	Person-years	HR (95%CI)	*p*	*N*	Person-years	HR (95% CI)	*p*	*N*	Person-years	HR (95% CI)	*p*
Per SD in concentration	744	1,551,468	0.945 (0.880–1.015)	0.119	1,234	1,978,052	0.951 (0.898–1.006)	0.082	869	1,127,639	0.895 (0.838–0.956)	0.001
**Quartiles**												
12.7–32.6	181	330,338	Ref		303	455,722	Ref		326	367,308	Ref	
32.6–46.8	152	352,207	0.802 (0.646–0.995)	0.045	320	498,491	0.963 (0.823–1.128)	0.643	228	316,920	0.826 (0.697–0.978)	0.027
46.8–62.0	199	391,839	0.941 (0.768–1.153)	0.559	309	516,263	0.887 (0.757–1.041)	0.142	187	257,300	0.841 (0.702–1.007)	0.059
62.0–104.0	212	477,084	0.812 (0.664–0.992)	0.042	302	507,576	0.869 (0.740–1.020)	0.086	128	186,111	0.789 (0.643–0.968)	0.023
			*p*_trend_ = 0.091				*p*_trend_ = 0.261				*p*_trend_ = 0.045	
**Category**												
Deficient (<25)	96	168,700	Ref		161	226,571	Ref		202	196,235	Ref	
Insufficient (25∼50)	274	596,057	0.833 (0.659–1.052)	0.125	531	843,323	0.887 (0.743–1.059)	0.184	401	551,175	0.724 (0.611–0.858)	<0.001
Optimal (> 50)	374	786,711	0.853 (0.680–1.071)	0.170	542	908,158	0.823 (0.689–0.983)	0.031	266	380,229	0.701 (0.583–0.843)	<0.001
			*p*_trend_ = 0.294				*p*_trend_ = 0.086				*p*_trend_ < 0.001	

*All models were adjusted for sex, age, income, education, smoking status, and vitamin D supplements.*

### Dose-Response Pattern Between 25OHD and Incident Psoriasis

**FIGURE 1:**
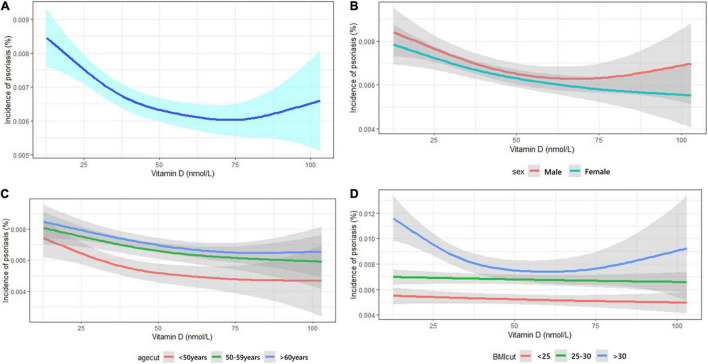
Dose-effect relationship between vitamin D and psoriasis.

The cubic splines show a non-linear relationship between 25OHD concentration and risk of incident psoriasis (Figure 1). The risk of incident psoriasis decreased when the circulating 25OHD concentration increased from 0 to 50 nmol/L, but thereafter the effect reduced. The analysis was also conducted by subgroups of sex, age, and BMI, and the effect of 25OHD was modified by obesity since the curves were almost linear in participants having BMI < 30 kg/m^2^.

### Mendelian Randomization Analysis for Genetically Determined 25OHD Level With Psoriasis Risk

**FIGURE 2:**
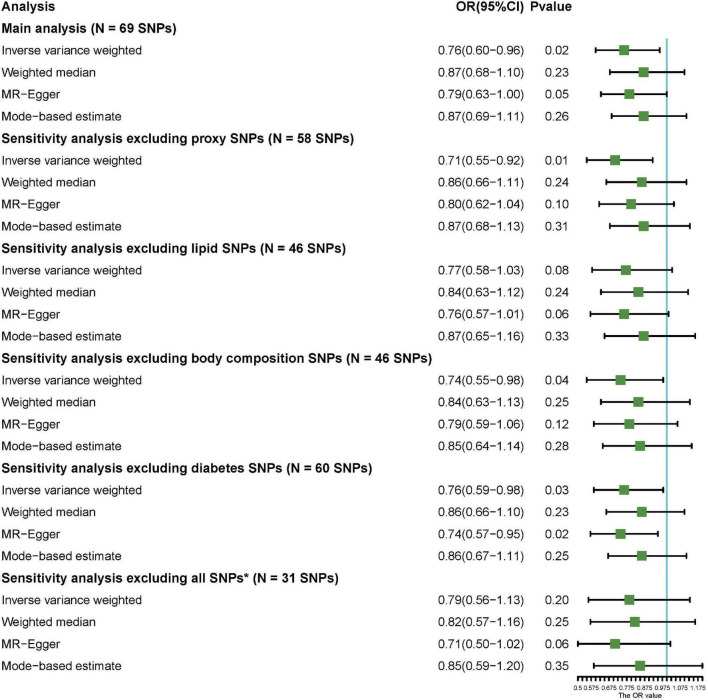
Results of Mendelian randomization analysis.

Sixty-nine common and conditionally independent SNPs (Supplementary Table 4) explaining 3.1% of the variation of circulating 25OHD levels were included for the MR analysis. We identified no significant associations with known potentially pleiotropic confounders influencing the exposure–outcome association based on PhenoScanner search, at a Bonferroni-correction threshold of 5 × 10^–8^. However, an enrichment in SNPs associated with certain trait categories, including body composition and serum lipid traits, was observed; and this may be associated with the risk of psoriasis. A detailed description of the SNP-trait association is provided in Supplementary Table 5. Among 462,933 participants in the cohort, one genetically predicted increment in the SD of 25OHD was associated with 24% decreased risk of psoriasis (OR_*IVW*_ = 0.76; 95% CI 0.60–0.96; *p* = 0.02) according to the IVW method. In addition, MR-Egger method suggested a significant association (OR_*MR*–*Egger*_ = 0.79; 95% CI 0.63–1.00; *p* = 0.05), while the weighted median and mode-based estimates presented with a comparable effect size (OR = 0.87) with no statistical significance. Additionally, sensitivity analysis was conducted to exclude proxy SNPs and covariate-associated SNPs, and the results also showed a causality between the genetically predicted level of 25OHD and risk of psoriasis after excluding proxy SNPs (OR_*IVW*_ = 0.71; 95% CI: 0.55–0.92; *p* = 0.01), body-composition associated SNPs (OR_*IVW*_ = 0.74, 95% CI: 0.55–0.98; *p* = 0.04), and diabetes-associated SNPs (OR_*IVW*_ = 0.76, 95% CI: 0.59–0.98; *p* = 0.03) (Figure 2). However, when all comorbidity-associated SNPs were excluded, the result changed to negative; but the trend still remains (OR_*IVW*_ = 0.79, 95% CI: 0.56–1.13; *p* = 0.20). A scatter plot of the MR analysis investigating the effect of 25OHD on psoriasis was presented in Supplementary Figure 2. Meanwhile, MR-PRESSO method was used to find evidence for pleiotropy (1,000 simulations). The MR estimates for psoriasis did not alter the conclusion after removing one outlier SNP with increased evidence of pleiotropic effects (Supplementary Table 6).

## Discussion

We first examined the association of circulating 25OHD level with the risk of incident psoriasis in a large-scale longitudinal data from the UK Biobank, and then investigated the causal relationship of genetically predicted level of 25OHD and psoriasis based on MR analysis. Individuals with 25OHD deficiency were more likely to have psoriasis, especially for obese subjects or aged under 50 years. This relationship was additionally confirmed by MR analysis.

Previously, a large number of observational studies tried to determine the association of circulating 25OHD with the risk of psoriasis, and most reported a lower serum level of 25OHD in patients with psoriasis compared to controls. A meta-analysis by Lee and Song found that psoriatic patients had a lower level of serum 25OHD compared to healthy controls, and revealed a small but significant correlation between circulating 25OHD level and the severity of psoriasis (22). Pitukweerakul et al. found the relationship between hypovitaminosis D and psoriasis, but did not establish a causal relationship (23). In the current study, we first used a large-scale prospective cohort and confirmed that 25OHD deficiency could increase the incidence of psoriasis in the following 11 years. A non-linear relationship between serum 25OHD concentration and the incidence of psoriasis was found. Results showed that the protective effect of vitamin D on incident psoriasis was quite obvious, but it seems to work under a specific threshold, which has not been reported in previous studies. However, we would refrain from suggesting that the high serum 25OHD concentration had opposite effect because of the large CI. The relatively small sample size of people with high serum 25OHD concentration might account for this result. The existence of the threshold and the probable mechanisms were still unclear, which need further confirmations.

The effect of 25OHD on incident psoriasis was also modified by obesity. The protective effect of vitamin D on incident psoriasis seem to be larger in obese individuals. This is consistent with previous finding that serum 25OHD was significantly associated with the risk of psoriasis only in patients with central obesity (24). These findings suggested that the effect of decreased 25OHD on the pathogenesis of psoriasis might be closely related to obesity, a well-known comorbidity of psoriasis. However, the role obesity played in the relationships and the specific associations among obesity, 25OHD, and psoriasis were seldom discussed in previous studies. Several hypotheses have been proposed such as the influence of metabolic disorders of obese patients, and there might be a mediating effect of serum 25OHD between obesity and psoriasis, but no further confirmation (24, 25). Previous evidences suggested that vitamin D also has beneficial effects on serum lipid profiles and thus cardiovascular health and other comorbidities (26, 27). Thus, it might suggest that vitamin D reduce the risk of psoriasis and other comorbidities at the same time and result in synergy effects in obese people with more complications. Meanwhile, as a fat-soluble vitamin, the absorption and metabolism of vitamin D might be affected by fat distribution (28). The changes of distribution and metabolic function of fat in obese people might directly or indirectly influence the effect of vitamin D. And these might be reasons that obesity modify the association between vitamin D and psoriasis. However, further studies are still needed to verify these hypotheses.

We used 69 common and conditionally independent SNPs from the newly published GWAS summary datasets of vitamin D in our MR analysis, and further confirmed the causal relationship between 25OHD with psoriasis at genetic level. The MR is an approach that uses genetic variants associated with a modifiable exposure or biological intermediate to estimate the causal relationship between these variables and a medically relevant outcome (29). The random allocation of genetic variants at conception reduces confounding from environmental factors and thus strengthens causal inference (30). Therefore, our results of MR further supported vitamin D deficiency as a possible cause of incident psoriasis. However, results of additional sensitivity analysis were negative after all SNPs associated with lipid, BMI, and diabetes were excluded, while the effect size did not change evidently. The reason might be the limitation of strength of these included SNPs, which explains less variation of circulating 25OHD levels. Further GWASs might be needed to expand the range of genetic instruments and obtain more reliable results.

25OHD is produced by the skin under the action of ultraviolet light and dietary intake. While topical 25OHD analogs exert various anti-inflammatory, anti-oxidant, and immunomodulatory actions by modulating innate and adaptive immune system, blocking proliferation, and promoting differentiation and maturation of keratinocyte is recommended as a treatment option by the National Psoriasis Foundation; the application of oral 25OHD supplementation as an adjunctive therapeutic options for psoriasis is still discussed, and its clinical use is predominantly based on experience of dermatologists and nutritionists (31). However, compared to patients with single psoriasis, patients of psoriasis with comorbidities such as psoriatic arthritis and obesity were more commonly recommend vitamin D supplementation (32). And it has been reported that this supplementation may be useful for comorbidity prevention in some cases (33). Previous studies that tried to determine the association between circulating 25OHD and psoriasis severity presented inconsistent findings. Mattozzi et al. showed that serum 25OHD level was significantly associated with the psoriasis area and severity index (PASI) score in patients with psoriasis (9). However, a randomized, double-blind, placebo-controlled trial (RCT) found that oral 25OHD3 supplementation did not significantly alleviate plaque psoriasis compared to the placebo (10). Another RCT conducted by the same research team found that monthly use of 25OHD3 supplementation did not reduce the severity of mild psoriasis compared to placebo as well (34). A small-scale RCT in Asians indicated clinical efficacy of oral 25OHD2 in the treatment of psoriasis without increasing adverse events (11). However, a recent meta-analysis study of RCTs did not support the efficacy of oral vitamin D supplementation in lessening the severity of psoriasis (35). Additionally, no studies confirmed the optimal dose of systemic 25OHD supplement in the treatment of psoriasis, although most of them did not observe adverse effects within a relatively narrow range of dosages (0.25–2 μg/d) (36). Combined with our finding, we deliberately concluded that 25OHD has a causal relationship with psoriasis, and this effect is modified by obesity. Cluster RCT studies in a general population with a high incidence rate of psoriasis would be warranted to confirm the effectiveness of systemic 25OHD supplementation in the prevention of psoriasis, especially for obese individuals with a deficient level of 25OHD, and to determine the optimal dose and adverse effects of the supplementation.

### Strengths and Limitations

This study has several strengths. First, the large sample size in addition to a prospective study design provide a higher level of evidence than retrospective studies. Second, a non-linear association and effect modification by obesity were identified, and this may explain some of the discrepancies among previous studies. Third, the MR analysis further enhanced our findings at genetic level, by constructing instrumental variables as a measure of exposure.

Nevertheless, this study also has limitations. The study population came from Europe, and the findings should be validated in different races and regions. Additionally, although some confounders were adjusted, unknown and unmeasured confounders and effect modifiers might be ignored. Also, the potential bias due to participant overlap have not been calculated because the conditions about the overlap of participants from these two published GWAS summary datasets could not been obtained. Last but not the least, the association of 25OHD and severity of psoriasis could not be estimated in the current study since relevant information such as PASI score was not available.

## Conclusion

This prospective cohort study shows that 25OHD was inversely associated with the risk of incident psoriasis, and effect modification by obesity was identified. MR analysis further confirmed the causality at genetic level.

## Data Availability Statement

Publicly available datasets were analyzed in this study. This data can be found here: https://biobank.ctsu.ox.ac.uk/crystal/index.cgi.

## Ethics Statement

The studies involving human participants were reviewed and approved by the North West Multi-Center Research Ethics Committee (UK biobank application ID #55257). The patients/participants provided their written informed consent to participate in this study.

## Author Contributions

YZ: conception, bibliographical research, manuscript development, and writing. DJ and GZ: bibliographical research, manuscript development, and writing. YX: bibliographical research and supervision of the manuscript. MS, XC, and HL: critical revision and supervision of the manuscript. All authors contributed to the article and approved the submitted version.

## Conflict of Interest

The authors declare that the research was conducted in the absence of any commercial or financial relationships that could be construed as a potential conflict of interest.

## Publisher’s Note

All claims expressed in this article are solely those of the authors and do not necessarily represent those of their affiliated organizations, or those of the publisher, the editors and the reviewers. Any product that may be evaluated in this article, or claim that may be made by its manufacturer, is not guaranteed or endorsed by the publisher.

## Footnotes

1
https://gwas.mrcieu.ac.uk/

## References

[1] Michalek I Loring B John S. A systematic review of worldwide epidemiology of psoriasis. *J Eur Acad Dermatol Venereol.* (2017) 31:205–12. 10.1111/jdv.13854 27573025

[2] Nestle F Kaplan D Barker J. Psoriasis. *N Engl J Med.* (2009) 361:496–509. 19641206 10.1056/NEJMra0804595

[3] Cai Y Fleming C Yan J. New insights of T cells in the pathogenesis of psoriasis. *Cell Mol Immunol.* (2012) 9:302–9. 10.1038/cmi.2012.15 22705915 PMC4132586

[4] Florek A Wang C Armstrong A. Treatment preferences and treatment satisfaction among psoriasis patients: a systematic review. *Arch Dermatol Res.* (2018) 310:271–319. 10.1007/s00403-018-1808-x 29442137

[5] Barrea L Savanelli M Di Somma C Napolitano M Megna M Colao A Vitamin D and its role in psoriasis: an overview of the dermatologist and nutritionist. *Rev Endocr Metab Disord.* (2017) 18:195–205. 10.1007/s11154-017-9411-6 28176237 PMC5486909

[6] Cantorna M. Vitamin D and autoimmunity: is vitamin D status an environmental factor affecting autoimmune disease prevalence? *Proc Soc Exp Biol Med.* (2000) 223:230–3. 10.1046/j.1525-1373.2000.22333.x 10719834

[7] Cyprian F Lefkou E Varoudi K Girardi G. Immunomodulatory effects of vitamin D in pregnancy and beyond. *Front Immunol.* (2019) 10:2739. 10.3389/fimmu.2019.02739 31824513 PMC6883724

[8] Karthaus N van Spriel A Looman M Chen S Spilgies L Lieben L Vitamin D controls murine and human plasmacytoid dendritic cell function. *J Investig Dermatol.* (2014) 134:1255–64. 10.1038/jid.2013.501 24352045

[9] Mattozzi C Paolino G Salvi M Macaluso L Scarnò M De Vita G Correlation between plasmatic levels of vitamin D and PASI score. *G Ital Dermatol Venereol.* (2018) 153:155–60. 10.23736/S0392-0488.17.05622-X 29144098

[10] Ingram M Jones M Stonehouse W Jarrett P Scragg R Mugridge O Oral vitamin D supplementation for chronic plaque psoriasis: a randomized, double-blind, placebo-controlled trial. *J Dermatol Treat.* (2018) 29:648–57. 10.1080/09546634.2018.1444728 29480035

[11] Disphanurat W Viarasilpa W Chakkavittumrong P Pongcharoen P. The clinical effect of oral vitamin D2 supplementation on psoriasis: a double-blind, randomized, placebo-controlled study. *Dermatol Res Pract.* (2019) 2019:5237642. 10.1155/2019/5237642 31139214 PMC6500602

[12] Trehearne A. Genetics, lifestyle and environment. UK Biobank is an open access resource following the lives of 500,000 participants to improve the health of future generations. *Bundesgesundheitsblatt Gesundheitsforschung Gesundheitsschutz.* (2016) 59:361–7. 10.1007/s00103-015-2297-0 26753864

[13] Lin L Smeeth L Langan S Warren-Gash C. Distribution of vitamin D status in the UK: a cross-sectional analysis of UK Biobank. *BMJ Open.* (2021) 11:e038503. 10.1136/bmjopen-2020-038503 33408196 PMC7789460

[14] Manousaki D Mitchell R Dudding T Haworth S Harroud A Forgetta V Genome-wide association study for vitamin D levels reveals 69 independent loci. *Am J Hum Genet.* (2020) 106:327–37. 10.1016/j.ajhg.2020.01.017 32059762 PMC7058824

[15] Hemani G Zheng J Elsworth B Wade K Haberland V Baird D The MR-Base platform supports systematic causal inference across the human phenome. *Elife.* (2018) 7:e34408. 10.7554/eLife.34408 29846171 PMC5976434

[16] Efstathiadou A Gill D McGrane F Quinn T Dawson J. Genetically determined uric acid and the risk of cardiovascular and neurovascular diseases: a mendelian randomization study of outcomes investigated in randomized trials. *J Am Heart Assoc.* (2019) 8:e012738. 10.1161/JAHA.119.012738 31438759 PMC6755826

[17] Lawlor D Harbord R Sterne J Timpson N Davey Smith G. Mendelian randomization: using genes as instruments for making causal inferences in epidemiology. *Stat Med.* (2008) 27:1133–63. 10.1002/sim.3034 17886233

[18] Burgess S Butterworth A Thompson S. Mendelian randomization analysis with multiple genetic variants using summarized data. *Genet Epidemiol.* (2013) 37:658–65. 10.1002/gepi.21758 24114802 PMC4377079

[19] Bowden J Davey Smith G Burgess S. Mendelian randomization with invalid instruments: effect estimation and bias detection through Egger regression. *Int J Epidemiol.* (2015) 44:512–25. 10.1093/ije/dyv080 26050253 PMC4469799

[20] Bowden J Davey Smith G Haycock P Burgess S. Consistent estimation in Mendelian randomization with some invalid instruments using a weighted median estimator. *Genet Epidemiol.* (2016) 40:304–14. 10.1002/gepi.21965 27061298 PMC4849733

[21] Hartwig F Davey Smith G Bowden J. Robust inference in summary data Mendelian randomization *via* the zero modal pleiotropy assumption. *Int J Epidemiol.* (2017) 46:1985–98. 10.1093/ije/dyx102 29040600 PMC5837715

[22] Lee Y Song G. Association between circulating 25-hydroxyvitamin D levels and psoriasis, and correlation with disease severity: a meta-analysis. *Clin Exp Dermatol.* (2018) 43:529–35. 10.1111/ced.13381 29341195

[23] Pitukweerakul S Thavaraputta S Prachuapthunyachart S Karnchanasorn R. Hypovitaminosis D is associated with psoriasis: a systematic review and meta-analysis. *Kansas J Med.* (2019) 12:103–8. 10.17161/kjm.v12i4.13255 31803350 PMC6884011

[24] Kuang Y Xiao Y Fang Z Zhang Y Shen M Chen X Association of serum vitamin D with psoriasis and effect modification by central obesity. *Front Med.* (2020) 7:236. 10.3389/fmed.2020.00236 PMC7315806 32626717

[25] Kanda N Hoashi T Saeki H. Nutrition and psoriasis. *Int J Mol Sci.* (2020) 21:5405. 10.3390/ijms21155405 PMC7432353 32751360

[26] Dibaba D. Effect of vitamin D supplementation on serum lipid profiles: a systematic review and meta-analysis. *Nutr Rev.* (2019) 77:890–902. 10.1093/nutrit/nuz037 31407792

[27] Jafari T Fallah A Barani A. Effects of vitamin D on serum lipid profile in patients with type 2 diabetes: a meta-analysis of randomized controlled trials. *Clin Nutr (Edinburgh Scotland).* (2016) 35:1259–68. 10.1016/j.clnu.2016.03.001 27020528

[28] Borel P Caillaud D Cano N. Vitamin D bioavailability: state of the art. *Crit Rev Food Sci Nutr.* (2015) 55:1193–205. 10.1080/10408398.2012.688897 24915331

[29] Evans D Davey Smith G. Mendelian randomization: new applications in the coming age of hypothesis-free causality. *Annu Rev Genomics Hum Genet.* (2015) 16:327–50. 10.1146/annurev-genom-090314-050016 25939054

[30] Georgakis M Gill D. Mendelian randomization studies in stroke: exploration of risk factors and drug targets with human genetic data. *Stroke.* (2021) 52:2992–3003. 10.1161/strokeaha.120.032617 34399585

[31] Megna M Ferrillo M Barrea L Patruno C Muscogiuri G Savastano S Vitamin D and psoriasis: an update for dermatologists and nutritionists. *Minerva Endocrinol.* (2020) 45:138–47. 10.23736/S0391-1977.20.03190-9 32340428

[32] Ford A Siegel M Bagel J Cordoro K Garg A Gottlieb A Dietary recommendations for adults with psoriasis or psoriatic arthritis from the medical board of the national psoriasis foundation: a systematic review. *JAMA Dermatol.* (2018) 154:934–50. 10.1001/jamadermatol.2018.1412 29926091

[33] Zuccotti E Oliveri M Girometta C Ratto D Di Iorio C Occhinegro A Nutritional strategies for psoriasis: current scientific evidence in clinical trials. *Eur Rev Med Pharmacol Sci.* (2018) 22:8537–51. 10.26355/eurrev_201812_16554 30556896

[34] Jarrett P Camargo C Coomarasamy C Scragg R. A randomized, double-blind, placebo-controlled trial of the effect of monthly vitamin D supplementation in mild psoriasis. *J Dermatol Treat.* (2018) 29:324–8. 10.1080/09546634.2017.1373735 28849682

[35] Theodoridis X Grammatikopoulou M Stamouli E Talimtzi P Pagkalidou E Zafiriou E Effectiveness of oral vitamin D supplementation in lessening disease severity among patients with psoriasis: a systematic review and meta-analysis of randomized controlled trials. *Nutrition.* (2021) 82:111024. 10.1016/j.nut.2020.111024 33183899

[36] Stanescu A Simionescu A Diaconu C. Oral vitamin D therapy in patients with psoriasis. *Nutrients.* (2021) 13:163. 10.3390/nu13010163 33419149 PMC7825555

