# Supplementary material for: Evidence of a Causal Relationship Between Vitamin D Status and Risk of Psoriasis From the UK Biobank Study
Source: Front Nutr. 2022 Jul 25;9:807344. doi: 10.3389/fnut.2022.807344 (PMC9359095; doi:10.3389/fnut.2022.807344)
Supplement: Supplementary file 1 [file Table_1.DOCX]

eTable 1. Association of vitamin D concentrations with incident psoriasis in different sex categories.

| Vitamin D concentration, nomol/L | Female | | | |  | Male | | | |
| --- | --- | --- | --- | --- | --- | --- | --- | --- | --- |
|  | N | Person-Years | HR (95%CI) ^a^ | *P* |  | N | Person-Years | HR (95%CI) ^b^ | *P* |
| Per SD in concentration | 1495 | 2511558 | 0.937 (0.890-0.986) | 0.013 |  | 1361 | 2162904 | 0.937 (0.888-0.988) | 0.017 |
| Quartiles |  |  |  |  |  |  |  |  |  |
| 12.7-32.6 | 425 | 619565 | Ref |  |  | 387 | 541183 | Ref |  |
| 32.6-46.8 | 366 | 629201 | 0.876 (0.761-1.008) | 0.065 |  | 337 | 542743 | 0.886 (0.765-1.026) | 0.107 |
| 46.8-62.0 | 372 | 625659 | 0.918 (0.798-1.058) | 0.238 |  | 325 | 542842 | 0.868 (0.747-1.007) | 0.063 |
| 62.0-104.0 | 332 | 637133 | 0.826 (0.712-0.957) | 0.011 |  | 312 | 536136 | 0.852 (0.731-0.992) | 0.040 |
|  |  |  | P_trend_=0.068 |  |  |  |  | P_trend_=0.140 |  |
| Category |  |  |  |  |  |  |  |  |  |
| Deficient (<25) | 238 | 317795 | Ref |  |  | 222 | 278400 | Ref |  |
| Insufficient (25~50) | 625 | 1070708 | 0.813 (0.699-0.945) | 0.007 |  | 585 | 927577 | 0.812 (0.695-0.949) | 0.009 |
| Optimal (>50) | 632 | 1123055 | 0.820 (0.704-0.956) | 0.011 |  | 554 | 956927 | 0.761 (0.649-0.893) | 0.001 |
|  |  |  | P_trend_=0.018 |  |  |  |  | P_trend_=0.003 |  |

^a^ Adjusted for age, BMI, income, education, smoking status, and vitamin D supplements;

^b^ Also adjusted for age, BMI, income, education, smoking status, and vitamin D supplements.
