# Supplementary material for: Evidence of a Causal Relationship Between Vitamin D Status and Risk of Psoriasis From the UK Biobank Study
Source: Front Nutr. 2022 Jul 25;9:807344. doi: 10.3389/fnut.2022.807344 (PMC9359095; doi:10.3389/fnut.2022.807344)
Supplement: Supplementary file 2 [file Table_2.DOCX]

eTable 2. Association of vitamin D concentrations with incident psoriasis in different age categories.

| vitamin D concentration, nmol/L | <50 years old | | | |  | 50~60 years old | | | |  | >60 years old | | |  |
| --- | --- | --- | --- | --- | --- | --- | --- | --- | --- | --- | --- | --- | --- | --- |
|  | N | Person-Years | HR (95%CI) ^a^ | *P* |  | N | Person-Years | HR (95%CI) ^b^ | *P* |  | N | Person-Years | HR (95%CI) ^c^ | *P* |
| Per SD in concentration | 569 | 1111476 | 0.913 (0.844-0.988) | 0.024 |  | 969 | 1551792 | 0.942 (0.885-1.003) | 0.064 |  | 1318 | 2011194 | 0.948 (0.896-1.003) | 0.062 |
| Quartiles |  |  |  |  |  |  |  |  |  |  |  |  |  |  |
| 12.7-32.6 | 200 | 335242 | Ref |  |  | 289 | 411338 | Ref |  |  | 323 | 414168 | Ref |  |
| 32.6-46.8 | 141 | 287133 | 0.850 (0.684-1.057) | 0.144 |  | 252 | 396361 | 0.947 (0.799-1.122) | 0.528 |  | 310 | 488450 | 0.850 (0.727-0.994) | 0.042 |
| 46.8-62.0 | 125 | 250760 | 0.916 (0.731-1.147) | 0.444 |  | 218 | 377105 | 0.883 (0.739-1.055) | 0.171 |  | 354 | 540636 | 0.895 (0.769-1.043) | 0.156 |
| 62.0-104.0 | 103 | 238341 | 0.822 (0.645-1.047) | 0.113 |  | 210 | 366988 | 0.894 (0.745-1.072) | 0.226 |  | 331 | 567940 | 0.812 (0.694-0.949) | 0.009 |
|  |  |  | P_trend_=0.335 |  |  |  |  | P_trend_=0.499 |  |  |  |  | P_trend_=0.056 |  |
| Category |  |  |  |  |  |  |  |  |  |  |  |  |  |  |
| Deficient (<25) | 130 | 181707 | Ref |  |  | 170 | 214102 | Ref |  |  | 160 | 200386 | Ref |  |
| Insufficient (25~50) | 235 | 499055 | 0.692 (0.558-0.858) | 0.001 |  | 426 | 680227 | 0.838 (0.701-1.003) | 0.053 |  | 549 | 819003 | 0.883 (0.740-1.054) | 0.169 |
| Optimal (>50) | 204 | 430714 | 0.753 (0.602-0.942) | 0.013 |  | 373 | 657463 | 0.791 (0.658-0.952) | 0.013 |  | 609 | 991805 | 0.842 (0.705-1.005) | 0.057 |
|  |  |  | P_trend_=0.003 |  |  |  |  | P_trend_=0.045 |  |  |  |  | P_trend_=0.161 |  |

^a^ Adjusted for sex, BMI, income, education, smoking status, and vitamin D supplements;

^b^ Also adjusted for sex, BMI, income, education, smoking status, and vitamin D supplements;

^c^ Also adjusted for sex, BMI, income, education, smoking status, and vitamin D supplements.
