# Supplementary material for: Evidence of a Causal Relationship Between Vitamin D Status and Risk of Psoriasis From the UK Biobank Study
Source: Front Nutr. 2022 Jul 25;9:807344. doi: 10.3389/fnut.2022.807344 (PMC9359095; doi:10.3389/fnut.2022.807344)
Supplement: Supplementary file 3 [file Table_3.DOCX]

eTable 3. Interaction Effect between BMI and Vitamin D.

|  | Coeffect ^a^ | *P* |
| --- | --- | --- |
| BMI * Vitamin D concentration | 0.995 | 0.481 |
| BMI category * Vitamin D category |  |  |
| Overweight * Insufficient | 1.066 | 0.667 |
| Obesity * Insufficient | 0.842 | 0.242 |
| Overweight * Optimal | 0.975 | 0.863 |
| Obesity * Optimal | 0.781 | 0.095 |

^a^ Adjusted by age, sex, BMI, income, education, smoking status, and vitamin D supplements.
