# Supplementary figures and images for: Evidence of a Causal Relationship Between Vitamin D Status and Risk of Psoriasis From the UK Biobank Study
Source: Front Nutr. 2022 Jul 25;9:807344. doi: 10.3389/fnut.2022.807344 (PMC9359095; doi:10.3389/fnut.2022.807344)

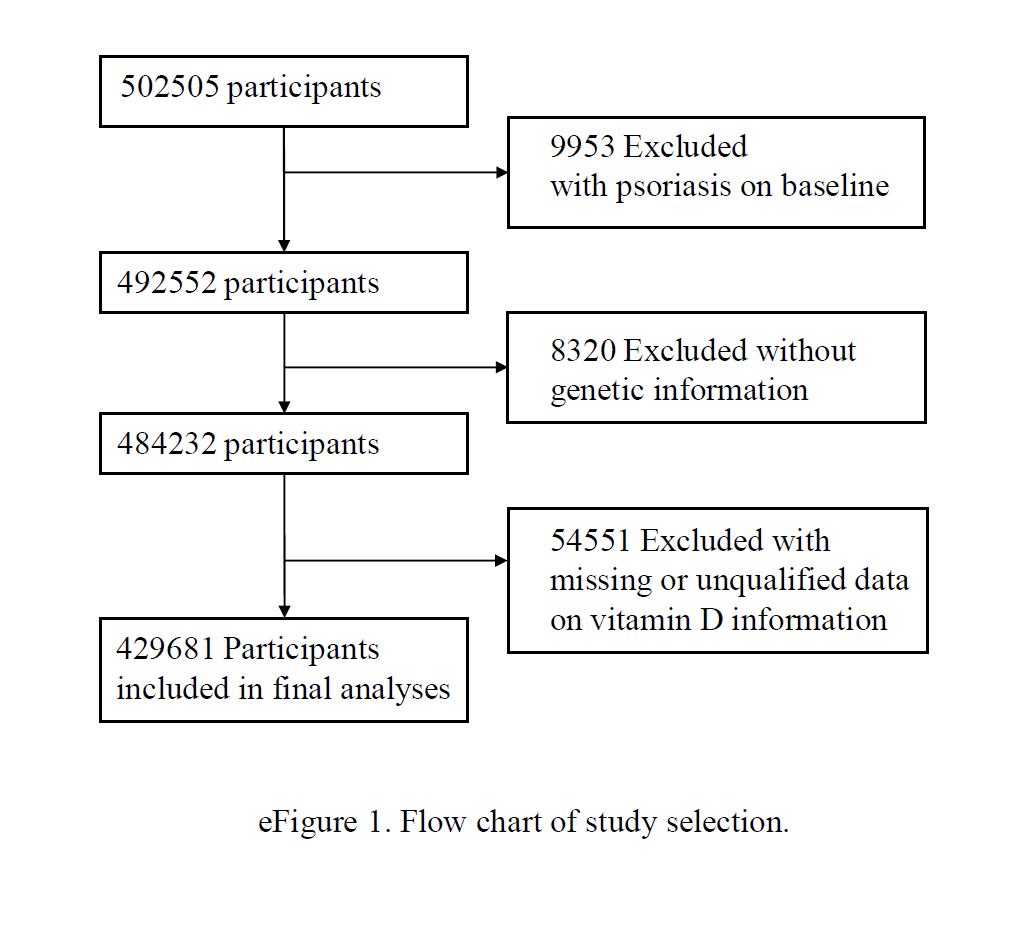

Supplement: Supplementary file 8 [file Image_1.png]

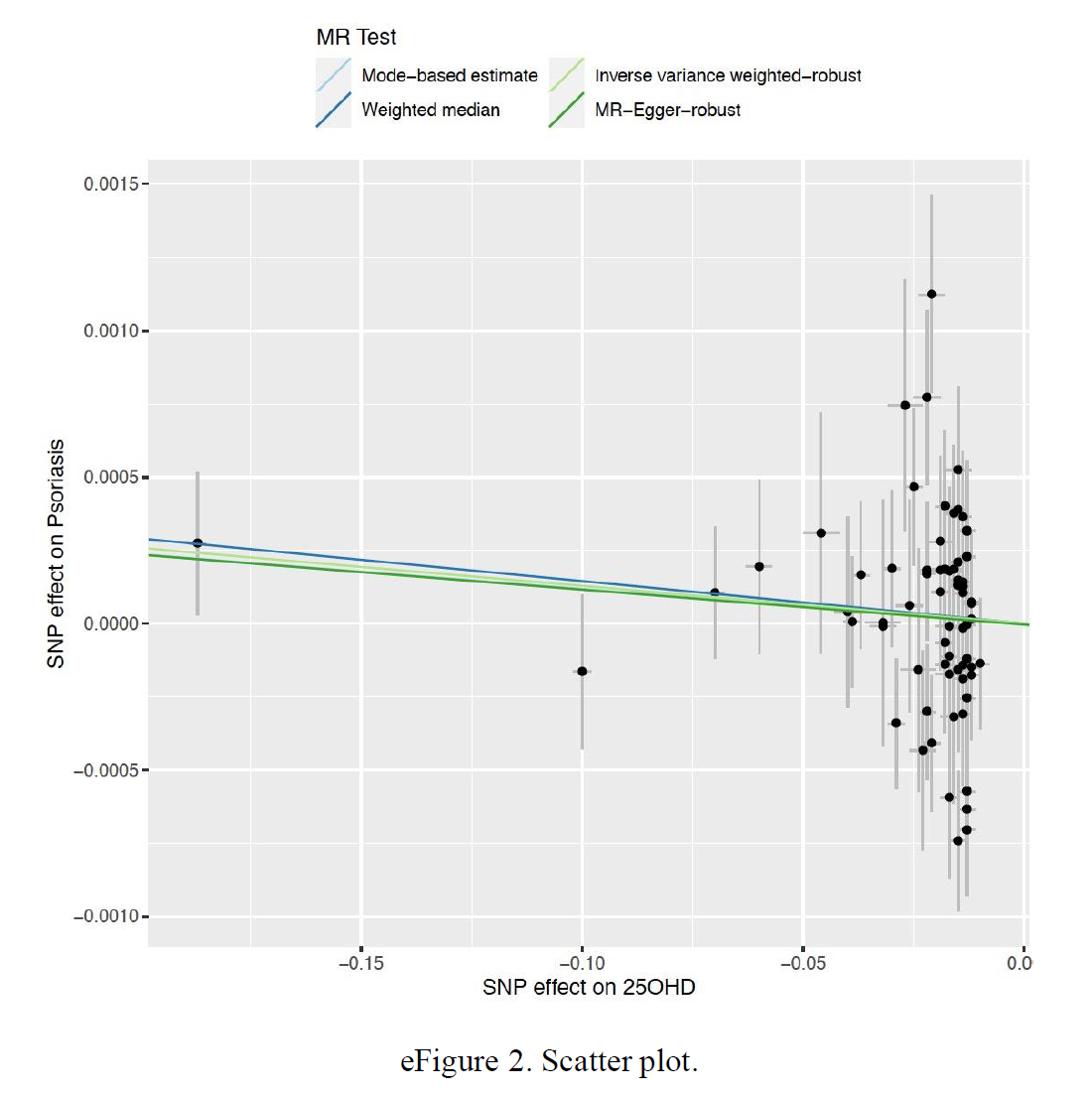

Supplement: Supplementary file 9 [file Image_2.png]
